# Genome wide association analysis for biomass related traits in common vetch (*Vicia sativa* L.)

**DOI:** 10.3389/fpls.2025.1647985

**Published:** 2025-09-29

**Authors:** Hui Jin, Jumei Zhang, Yordan Dimtrov, Xue Yang, Ruonan Du, Yu-e Wu, Danna Chang, Rui Zhang, Haibin Zhao

**Affiliations:** ^1^ Institute of Forage and Grassland Sciences, Heilongjiang Academy of Agricultural Sciences, Harbin, China; ^2^ Institute of Agricultural Resources and Regional Planning, Chinese Academy of Agricultural Sciences, Beijing, China

**Keywords:** association analysis, dry weight, fresh weight, gmarker-assisted selection (MAS), Vicia sativa L

## Abstract

**Introduction:**

Common vetch (*Vicia sativa* L.) is a leguminous plant widely cultivated in Asia, Europe, and the Americas, primarily used as a forage crop and green manure.

**Materials and methods:**

To identify loci significantly associated with biomass related traits, a genome-wide association study (GWAS) was perdormed in 172 common vetch accessions mainly from China and Europe. The single nucleotide polymorphisms (SNPs) were obtained through re-sequencing, while five biomass related traits, including the fresh weight (FW), dry weight (DW), fresh weight per plant (PFW), dry weight per plant (PDW) and plant height (PH), were evaluated across four environments.

**Results:**

In total, 33 loci were identified, and explain 11.6-22.5% of the phenotypic variances. Notably, among them, *qPDW1.2* and *qPFW1.3*, *qPH2.1* and *qFW2.1*, *qDW4.1* and *qFW4.1*, *qPH5.1* and *qPH5.1*, *qDW6.1* and *qFW6.1* are overlapped or located in the same genomic region, exhibiting pleiotropic effects on two traits, and should be prioritized in further vetch breeding programs. Additionally, all the 33 loci are novel compared with previous reports. Fifteen candidate genes for biomass related traits were identified for biomass related traits, encoding ethylene-responsive transcription factors, ABC transporter family proteins, serine/threonine-protein phosphatase, cellulose synthase, and auxin-responsive proteins. Furthermore, we successfully developed and verified a kompetitive allele-specific PCR (KASP) marker (*Kasp-DW1.1*) for dry weight. Accessions with more favorable alleles, superior biomass related traits and agronomic traits, eg. GLF295, GLF299, GLF313, GLF321, GLF325, GLF328, GLF329, GLF330, GLF331, GLF335, HZMC1382 and HZMC1487 are recommended as parental lines for further common vetch breeding.

**Discussion:**

This study enhances our understanding of the genetic architecture of biomass related traits and provides available novel loci, molecular tools and outstanding varieties for improving common vetch biomass in future breeding.

## Introduction

Common vetch (*Vicia sativa* L.), an important forage crop, native to the Mediterranean coast and West Asia (Hanelt and Mettin, 1989; Navrátilová et al., 2003; Xi et al., 2022). Common vetch serves multiple purposes, including forage and ecological restoration: 1) high-quality forage: with a crude protein content of 18-22%, it is an excellent feed for cattle and sheep (Meydani et al., 2014; De la Rosa et al., 2020; Nguyen et al., 2020); 2) ecological benefits: exhibits strong nitrogen-fixing capacity, improving soil fertility. Therefore, improving biomass-related traits is a key objective for common vetch (Barroso et al., 2025). It is now widely distributed in temperate and subtropical regions worldwide, including Europe, North America, Asia, and Oceania. Common vetch covers approximately 0.54 million hectares globally, with Russia, China, and Australia being the major producers (Çeliktaş et al., 2006; Tenopala et al., 2012; Jia et al., 2021). Introduced to China in the 1950s, common vetch is primarily cultivated in the northwest (Gansu, Qinghai), north (Inner Mongolia, Shanxi), and high-altitude regions of southwest China, with a total planting area of about 500,000 hectares (Mao et al., 2015; Dong et al., 2017; Jia et al., 2021; Xi et al., 2022). Typical yields include 6–15 tons/ha of hay and 1.2-2.5 tons/ha of seeds. It performs exceptionally well on the Qinghai-Tibet Plateau, serving as a crucial winter forage crop.

Now, nearly 500 common vetch accessions conserved in China national gene bank. Common vetch is diploid (2n=12), with a genome size of ~1.3 Gb. Molecular markers based on DNA sequence variations, primarily including simple sequence repeats (SSR) or single nucleotide polymorphism (SNP) (Liu et al., 2016), and Insertion-deletion (InDel). Compared to traditional markers, molecular markers offer the advantages of high abundance and environmental independence, enabling precise reflection of genomic differences (Chen et al., 2024; Rasheed et al., 2025). The widespread adoption of next-generation sequencing (NGS) technologies has made SNP the mainstream choice due to their high density and high-throughput detection capability, making them suitable for large-scale genetic analysis. In 2022, the release of a chromosome-level reference genome for *Vicia sativa* L. enabled SNP discovery cost-effective using NGS (Xi et al., 2022).

Marker assisted selection (MAS) utilizes molecular markers closely linked to target genes for early-stage selection, significantly shortening the breeding cycle. For instance, MAS breeding for the rice bacterial blight resistance gene *Xa23* tripled the efficiency of developing resistant lines (Wang et al., 2015). A typical MAS pipeline often begins with the identification of candidate genomic regions associated with desirable traits, followed by the development and validation of reliable markers, and finally, the integration of these markers into breeding programs. In practice, this sequence is commonly implemented as: (1) genome-wide association studies (GWAS) to identify candidate regions, (2) development of kompetitive allele-specific PCR(KASP) markers (Breseghello and Sorrells, 2006), and (3) application of MAS breeding. For example, in soybean oil content improvement, GWAS first identified the *GmDGAT1* gene, which was followed by the design of KASP markers (Breseghello and Sorrells, 2006), ultimately enabling the development of high-oil varieties (Li et al., 2013). GWAS plays a pivotal role in MAS by statistically analyzing the association between large numbers of molecular markers (>100,000 SNPs) and corresponding phenotypic traits to identify genomic regions responsible for target traits (Liu et al., 2017). A major advantage of GWAS lies in its ability to bypass the need for segregating populations by leveraging historical recombination events in natural populations, often achieving single-gene resolution (Liu et al., 2017). The rapid advancement of resequencing technologies has further accelerated GWAS research by enabling the discovery of vast numbers of SNPs within a short time frame, enhancing the study of complex traits in crops (Liu et al., 2017, 2019; Yang et al., 2020; Chen et al., 2024). Once significant loci are identified through GWAS, they can be effectively translated into breeding tools by converting SNPs into user-friendly KASP markers (Liu et al., 2016; Rasheed et al., 2025). KASP is a high-throughput, low-cost genotyping technology known for its speed, reliability, and wide application across major crops like rice, wheat, and maize (Rasheed et al., 2025). However, its application in certain crops, such as common vetch, has not yet been reported. The conversion of significant SNPs into KASP markers for field-deployable, high-throughput genotyping.

In the present study, we evaluated 5 biomass related traits in a diverse panel of 172 elite common vetch accessions for GWAS to (1) identify SNPs significantly associated with biomass related traits, (2) search for candidate genes of biomass yield traits for further study, and (3) develop available KASP markers for common vetch MAS breeding.

## Materials and methods

### Plant materials

**Figure 1:**
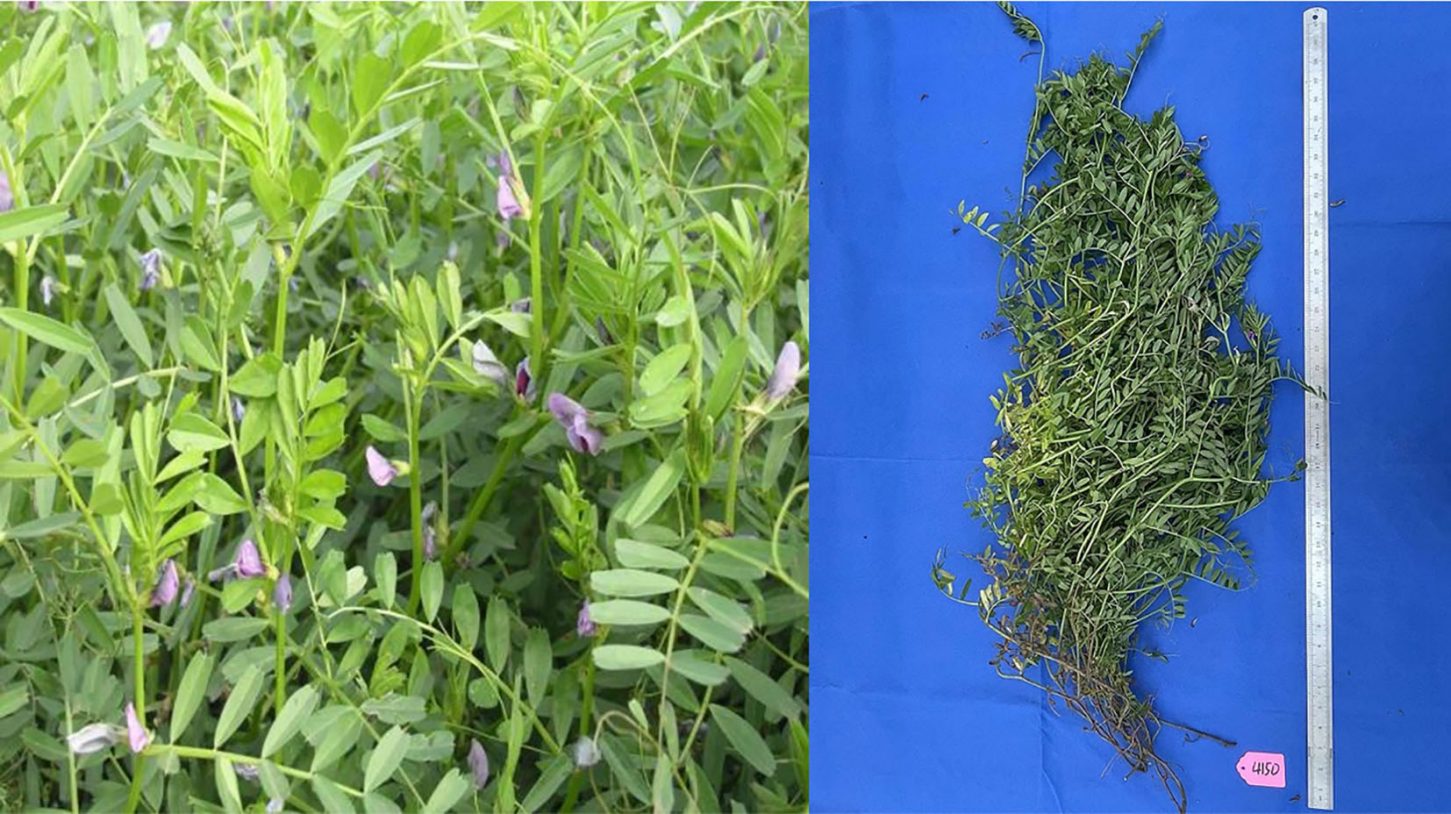
The 172 common vetch accessions in Heilongjiang.

We collected 172 common vetch accessions from 18 countries, with the largest proportion originating from China (108 accessions, 62.8%), followed by Russia (29, 16.9%), Ukraine (6, 3.5%), Spain (5, 2.9%), Belarus (4, 2.3%), Australia (3, 1.7%), Czech Republic (3, 1.7%), Poland (2, 1.2%), Germany (2, 1.2%), Sweden (2, 1.2%), and one accession each from Bulgaria, France, Greece, Italy, Lithuania, Mexico, Philippines, and Romania (**Supplementary Table S1**, **Figure 1**). The Chinese accessions were provided by the National Crop Germplasm Resources Green Manure Medium-term Storage Facility. All accessions were cultivated from 2020 to 2023 in Harbin, Heilongjiang Province (45°51’N, 126°45’E). Sowing occurred in mid-April each year, with harvesting in late July. A randomized block design was implemented, with each plot planted in three replicates. Each plot consisted of five rows of 5 meters long each, with a row spacing of 0.65 meters, and 100 seeds per row.

Biomass-related traits, including fresh weight (FW), dry weight (DW), fresh weight per plant (PFW), dry weight per plant (PDW), and plant height (PH), were evaluated following the methods described by Cao et al. (2007). PH was measured as the natural height from the base to top of the main stem. FW represents the fresh biomass yield per unit area, while DW indicates the dry biomass yield per unit area. Similarly, PFW refers to the fresh biomass yield per plant, and PDW denotes the dry biomass yield per plant. During the full flowering stage, 10 plants were randomly selected from each plot to measure PH. Additionally, three 1 m sample sections (excluding border rows) were randomly chosen from each plot to determine FW by averaging the fresh weight. The sampled fresh biomass was first placed in a 105°C oven for 30 min to deactivate enzymes and then dried at 70°C until a constant weight was achieved to record DW.

### Re-sequencing and data quality assessment

Whole-genome resequencing of 172 common vetch accessions was performed using PE150 sequencing, generating a total of 3,766.95Gb of raw data. After quality filtering, 3,727.56Gb of clean data were retained, yielding a high effective rate of 98.95%. The sequencing data exhibited excellent quality, with low error rates (0.03%) and high base accuracy, as reflected by Q_20_ (97.19%) and Q_30_ (92.03%) scores. The clean data were processed through a standardized bioinformatics pipeline: clean reads were aligned to the common vetch reference genome (GCF_026540005.1) using the BWA (v0.7.8) (Li and Durbin, 2009) and converted to binary alignment format via SAMtools (v0.1.19) (https://sourceforge.net/projects/samtools/files/samtools/0.1.19/). The average GC content was 35.70%, consistent with expectations for the species. Alignment to the reference genome achieved a high mapping rate of 99.03%. The average sequencing depth across samples was 15.18×, ensuring reliable variant detection. Genome coverage analysis revealed that 72.54% of the reference genome was covered at ≥1× depth, while 62.93% achieved ≥4× depth. SNP calling was performed with GATK HaplotypeCaller (v4.0.10.1) (Brouard et al., 2019). The SNPs were filtered by minor allele frequency (MAF) <0.05 and missing rate >10% (**Supplementary Table S2**) for further association mapping. These results confirm the high quality and reliability of the resequencing data, providing a solid foundation for subsequent GWAS analysis and MAS breeding of common vetch.

### Population analysis and GWAS

Population structure was assessed using ADMIXTURE (v1.3.0). Principal component analysis (PCA) and neighbor-joining trees were constructed using TASSEL v5.0 (Bradbury et al., 2007). To ensure the reliability of our results, stringent genetic background control measures were implemented in the mixed linear model (MLM) to minimize spurious associations (Zhu et al., 2021; Ma et al., 2022; Sun et al., 2024). Broad-sense heritability (*H_b_* ²) was calculated as *H*² = σ²*_g_*/(σ²*_g_* + σ²*_ge_*/n + σ²*_e_*/nr), where σ²*_g_* represents genetic variance, σ²*_ge_* genotype-environment interaction variance, and σ²e residual variance, with n environments and r replicates. GWAS was performed using a mixed linear model (MLM) in TASSEL v5.0, incorporating kinship matrices and principal components as covariates. In this study, the Bonferroni-Holm correction for multiple testing (*alpha* = 0.05) was too conservative and no significant MTAs were detected. Thus, markers with an adjusted - log_10_ (*P* value) = 5.0 were regarded as significantly associated (He et al., 2019; Rathan et al., 2022).

### Development and validation of KASP molecular markers

KASP primers were designed for trait-associated SNPs according to Chen et al., 2024. The reaction mixture contained 46 μL sterile distilled water, 12 μL tailed primer (100 μM), and 30 μL common primer (100 μM). Thermal cycling conditions included initial denaturation at 95°C for 15 min, followed by 10 touchdown cycles (94°C for 20s; annealing from 65°C, decreasing by 0.8°C/cycle for 25s) and 10 amplification cycles (94°C for 20s; 55°C for 60s).

### qRT-PCR experimental validation

Nineteen candidate genes linked to biomass related traits of common vetch were selected for expression profiling. High-bulk and low-bulk were established using accessions with extreme trait values. For PH, stem tip meristem tissue was collected from common vetch plants during the rapid stem elongation stage to perform qRT-PCR. For PDW, DW, PFW and FW, use stem tissues from common vetch plants during the flowering to pod-filling stages for qRT-PCR analysis targeting biomass accumulation genes. cDNA was synthesized using HiScript II, and gene-specific primers were designed with Primer 5.0. Quantitative PCR (20 μL total volume) included 2 μL cDNA, 10 μL SYBR Green master mix (ChamQ Universal), and 0.4 μL of each primer (10 μM). Technical triplicates were analyzed, and relative expression was quantified using the 2^^−ΔΔCt^ method with *actin* as the reference gene.

## Results

### Whole-genome resequencing of 172 common vetch accessions

Totally, 4,796,342 high-quality SNPs distributed across all 6 chromosomes were identified by whole-genome resequencing of 172 common vetch accessions, with an average density of 2,904.6 SNPs/Mb (**Table 1**; **Supplementary Table S2**). The genome-wide SNP distribution revealed substantial variations in marker density across tall the whole genome. Among these, chromosome 1 exhibited the highest SNP abundance with 1,040,667 identified variants spanning 324.8 Mb, yielding a marker density of 3,204.4 SNPs/Mb. Chromosome 2 followed closely with 1,011,653 SNPs across a similar physical length (324.6 Mb), resulting in a slightly lower density of 3,117.0 SNPs/Mb. Chromosome 4 demonstrated notable SNP clustering, containing 923,806 variants within a relatively compact 290.1 Mb physical interval, achieving the second-highest density (3,184.4 SNPs/Mb). In contrast, Chromosome 3 displayed reduced polymorphism with 679,356 SNPs across 290.7 Mb (2,337.1 SNPs/Mb), while chromosome 5 showed moderate density (2,613.9 SNPs/Mb) from 712,272 SNPs distributed over 272.5 Mb. Chromosome 6 emerged as an outlier, hosting 428,588 SNPs within the shortest physical interval (148.7 Mb), yet maintaining a relatively high density of 2,882.7 SNPs/Mb. Collectively, the 4,796,342 high-quality SNPs covering a total physical distance of 1,651.3 Mb with an average density of 2,904.6 SNPs/Mb.

**Table 1:** The SNPs used for the GWAS of biomass related traits in common vetch.

Chromosome	Number of SNP	Physical interval (Mb)	Density (marker/Mb)
1	1040667	324.8	3204.4
2	1011653	324.6	3117.0
3	679356	290.7	2337.1
4	923806	290.1	3184.4
5	712272	272.5	2613.9
6	428588	148.7	2882.7
all	4796342	1651.3	2904.6

### Population structure analysis of the 172 accessions

**Figure 2:**
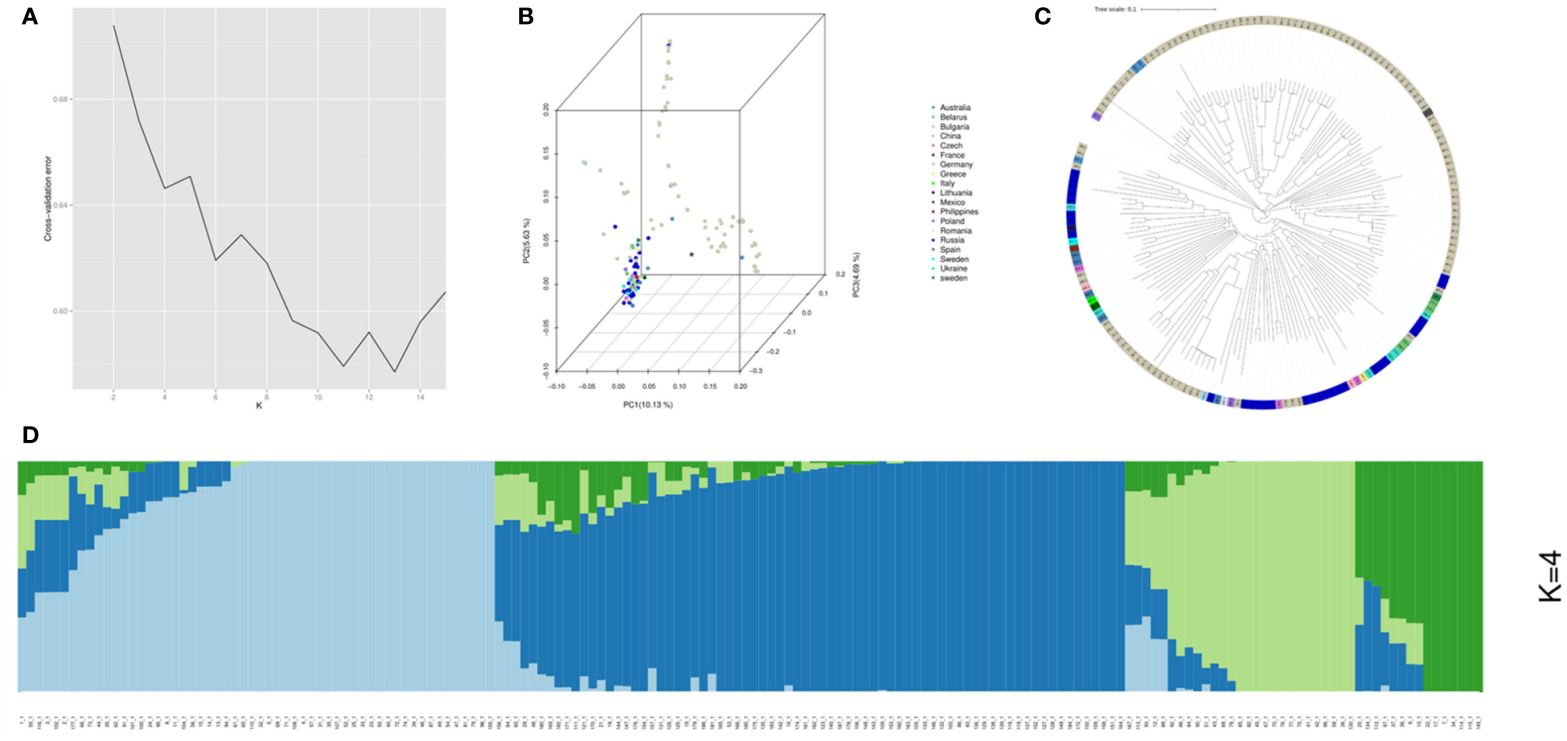
Population structure analysis for the 172 common vetch accessions. **(A)** The cross-validation error (CV error) for population structure analysis was assessed, with the lowest error (CV error = 0.62) observed at K=4. **(B)** Principal component analysis (PCA) was performed on the 172 common accessions. **(C)** A Neighbor-Joining tree was constructed for the 172 common vetch accessions. **(D)** Population structure analysis was conducted for the diverse panel.

Analysis of the 172 common vetch accessions revealed four distinct subpopulations (Pop1-Pop4). Among these, the POP1 comprising 95 accessions primarily from China, Russia, and Europe, suggesting broad adaptability. POP2, consisting of 50 accessions, was predominantly Chinese, indicating localized genetic homogeneity. POP3 (15 accessions) and POP4 (12 accessions) were also largely from China but included rare accessions from Europe and Australia, hinting at potential genetic isolation or unique adaptations. Phylogenetic and principal component analysis (PCA) validated these groupings (**Figure 2**).

Significant differences among sub-groups were observed for several parameters. Sub-group 1 exhibited the tallest PH (113.4 cm), significantly greater than Sub-group 4 (78.7 cm). While Sub-group 1 also produced the highest plant fresh weight (198.8 g), significantly outperforming other sub-groups. Notably, no significant differences were detected in PDW among any sub-groups. For FW and DW, Sub-group 1 (36.4 t/hm² and 6.3 t/hm²) yielded significantly more than Sub-groups 2, 3, and 4, which did not differ significantly from each other (**Supplementary Table S3**).

### Phenotype analysis and GWAS of biomass related traits in common vetch

**Figure 3:**
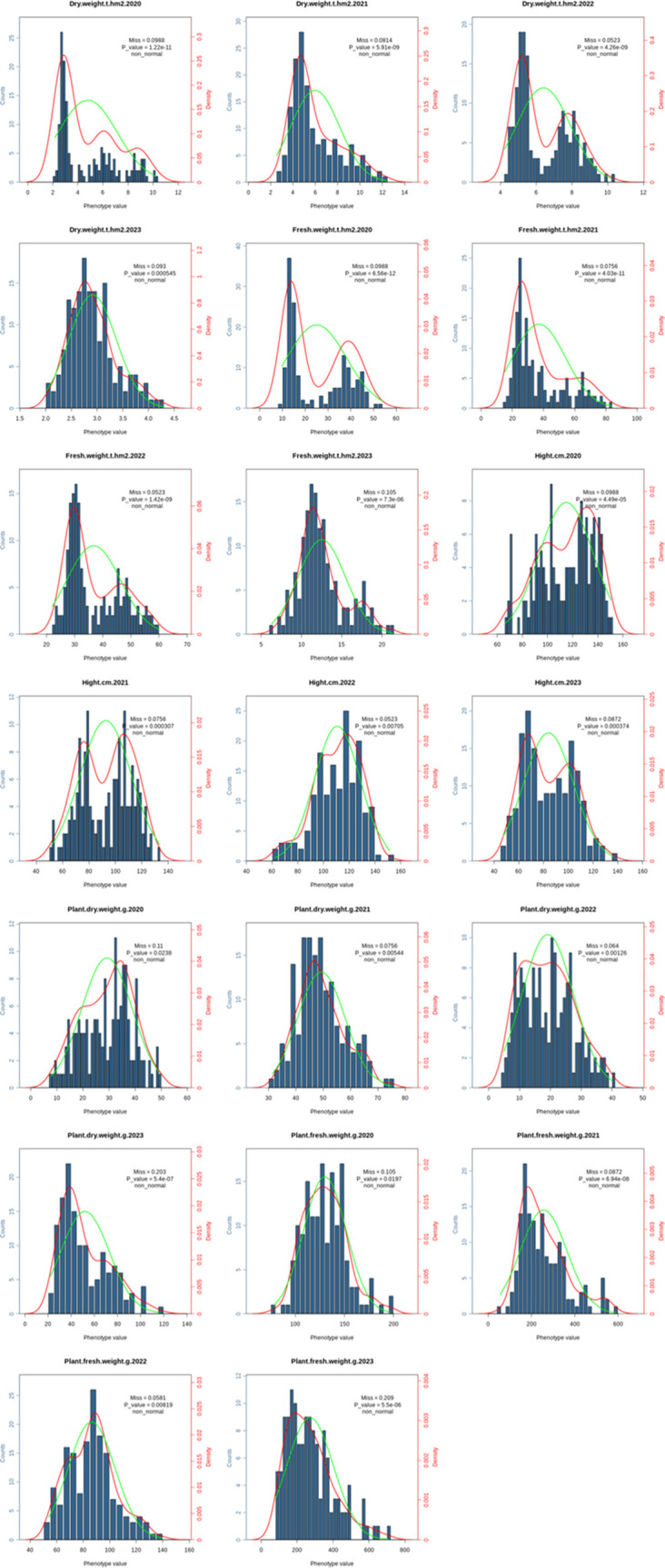
The phenotype distribution of the 5 biomass-related traits in 172 common vetch accessions.

Across four environments, 172 common vetch accessions were assessed for 5 biomass related traits. The average PH for the 172 common vetch accessions was 101 cm, with a standard deviation (SD) of 18.2 cm and a coefficient of variation (CV) of 0.181. In addition, the average PFW was 184.0 g with SD of 47.0 g and CV of 0.255, while the average PDW was 36.9 g with SD of 6.36 g and CV of 0.172. For biomass production, the average FW was 28.0 t·hm^-^² (SD = 9.92 t·hm^-^²; CV = 0.353), and the average DW was 5.05 t·hm^-^² with SD of 1.48 t·hm^-^² and CV of 0.293 (**Figure 3**). ANOVA revealed significant effects (*P <*0.01) of genotypes, environments, and genotype × environment interactions on each biomass related trait (**Supplementary Table S4**). The *H*_b_^2^ estimated for PH, PFW, PDW, FW and DW were 0.68, 0.65, 0.65, 0.62 and 0.63, respectively. PFW, FW and DW showed significant (*P <*0.01) and positive correlations with PH (*r* = 0.28, 0.74 and 0.70), and the FW showed a significant (*P <*0.01) and positive correlation with DW (*r*=0.97).

GWAS was conducted on 172 common vetch accessions across four environments. For DW, 7 significant loci were identified on chromosomes 1, 2, 4, 5, and 6, explaining 11.6% to 17.1% of the phenotypic variances (PVEs), with the highest contribution from *qDW4.3*. For FW, seven associated loci were found on chromosomes 1, 2, 4, 5, and 6, with PVEs ranging from 11.9% to 22.5%. Additionally, *qDW4.3* is the genetic locus with the largest span, approaching 23.1 Mb. Haplotype analysis indicates that this genetic region forms a single haplotype block. The results also suggest that the *qDW4.3* segment has strong linkage, residing in an area with fewer recombination events, which may indicate the presence of important quality traits or other significant characteristics in this region.

**Figure 4:**
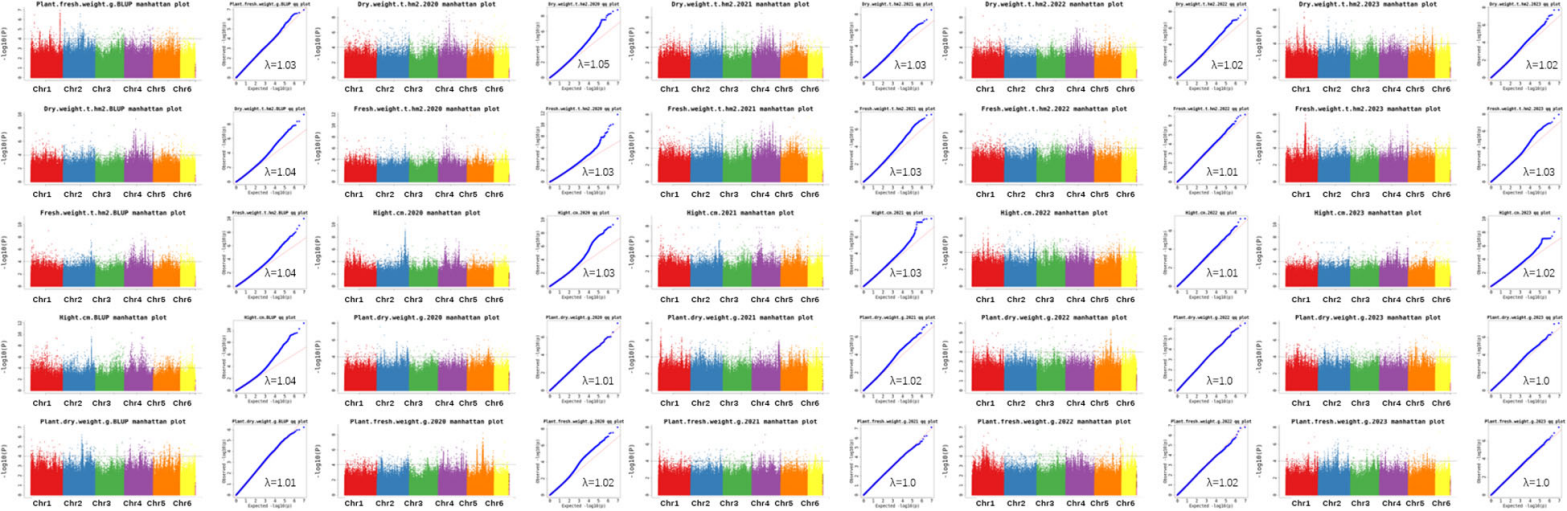
The Manhattan and Q-Q plot for the 5 biomass related traits in 172 common vetch accessions.

Notably, *qFW5.1* accounted for the maximum PVE (22.5%). Four significant loci for PDW were identified on chromosomes 1, 4, and 5, with PVEs ranging from 11.6% to 14.9%. Similarly, seven loci significantly associated with PFW were distributed across chromosomes 1, 2, and 4, explaining 11.6% to 16.8% of the PVEs. PH displayed the most extensive genomic distribution, with nine loci spanning chromosomes 1, 2, 3, 4, 5, and 6, explaining 11.6% to 14.9% of the PVEs (**Table 2**; **Figure 4**). In addition, several loci with multi-effects were identified, demonstrating their influence on multiple traits. For example, *qPDW1.2* and *qPFW1.3* were co-located at 285.8-292.8 Mb on chromosome 1. Similarly, *qPH2.1* and *qFW2.1* were co-located at 283.4-285.6 Mb on chromosome 2. On chromosome 4, *qDW4.1* and *qFW4.1* were co-located at 83.0-83.9 Mb, while *qDW6.1* and *qFW6.1* were co-located at 5.4-7.7 Mb on chromosome 6.

**Table 2:** The loci identified by the GWAS of 5 biomass related traits in 172 common vetch accessions.

QTL	Trait	Chromosome	Physical interval (Mb)	*P*-value	R^2^ (%)
*qDW1.1*	Dry weight	1	185.0-185.2	7-7.7	11.6-14
*qDW2.1*	Dry weight	2	106.8-111.0	7.1-8.4	12-15.3
*qDW4.1*	Dry weight	4	83.8-83.8	7.4-8.8	13.1-16.5
*qDW4.2*	Dry weight	4	94.3-96.1	7.2-8.6	12.4-16.5
*qDW4.3*	Dry weight	4	112.3-135.4	7-9.3	11.6-17.1
*qDW5.1*	Dry weight	5	110.2-118.7	7-7.7	11.6-14
*qDW6.1*	Dry weight	6	5.4-7.7	8.2-8.3	15.6-15.3
*qFW1.1*	Fresh weight	1	15.2-16.2	7.2-7.5	12.5-13.5
*qFW1.2*	Fresh weight	1	195.8-196.4	7.1-7.5	12-13.5
*qFW2.1*	Fresh weight	2	284.4-284.6	7.1-8.7	11.9-16.5
*qFW4.1*	Fresh weight	4	83.0-83.9	7.5-7.8	13.6-14
*qFW4.2*	Fresh weight	4	209.6-212	7.1-7.1	11.9-11.9
*qFW5.1*	Fresh weight	5	28.4-28.4	7.7-11.8	14.2-22.5
*qFW6.1*	Fresh weight	6	5.4-7.7	8.6-8.8	16.3-16.5
*qPDW1.1*	Plant dry weight	1	25.6-26.7	7.1-7.3	11.9-13
*qPDW1.2*	Plant dry weight	1	285.8-292.8	7-7.5	11.6-13.5
*qPDW4.1*	Plant dry weight	4	251.9-253.4	7.1-7.6	12-13.8
*qPDW5.1*	Plant dry weight	5	159.1-161.8	7-8.2	11.7-14.9
*qPFW1.1*	Plant fresh weight	1	6.1-7.2	7-8.5	11.7-16
*qPFW1.2*	Plant fresh weight	1	75.7-76.3	7.2-8	12.5-14.2
*qPFW1.3*	Plant fresh weight	1	286.8-291.8	8.5-8.9	15.9-16.8
*qPFW2.1*	Plant fresh weight	2	2.6-3.1	7-8.5	11.6-16
*qPFW2.2*	Plant fresh weight	2	22.8-23.1	7.2-7.9	12.5-14.2
*qPFW4.1*	Plant fresh weight	4	33.1-36.2	7-7.5	11.6-13.5
*qPFW4.2*	Plant fresh weight	4	227.7-232.3	7.3-7.6	12.8-13.8
*qPH1.1*	Plant height	1	82.2-83.5	7.3-8.2	12.8-14.9
*qPH2.1*	Plant height	2	283.4-285.6	7-7.6	11.7-13.8
*qPH3.1*	Plant height	3	175.2-178.2	7.1-8.2	11.9-14.9
*qPH3.2*	Plant height	3	213.8-216.9	7.2-7.3	12.6-12.8
*qPH4.1*	Plant height	4	51.2-53.5	7.2-7.5	12.5-13.5
*qPH4.2*	Plant height	4	201.7-204.8	7.5-7.5	13.6-13.5
*qPH5.1*	Plant height	5	238.7-241.2	7.1-7.4	12-13.2
*qPH6.1*	Plant height	6	144.3-146.3	7.3-7.6	12.8-13.8

### Identification and functional analysis of candidate genes

To identify high-confidence candidate genes, loci with stable and significant effects were analyzed based on genetic regions, gene annotations, and functional networks in model species. Totally, 19 candidate genes associated with common vetch biomass were identified, each playing diverse functional roles (**Supplementary Tables S5**-**S7**). Several genes were found to be associated with critical proteins. For example, *jg20144* (*qPH5.1*), *jg53159* (*qPH3.1*), and *jg38331* (*qFW1.1*) were encoded the ABC transporter family proteins, which facilitate the movement of various substrates across cellular membranes. Similarly, *jg28336* (*qPFW4.1*) is associated with cellulose synthase A catalytic subunit, a key enzyme in cellulose synthesis, while *jg36211* (*qPFW4.2*) corresponds to protein cellulose synthase interactive 3, a component involved in cell wall formation. Other significant genes include *jg54309* (*qPH3.2*), which is the production of F-box proteins that regulate protein degradation and signaling pathways, and *jg5441* (*qPFW2.2*), which was encoded to serine/threonine-protein phosphatase 7 long-form homolog, a modulator of cellular processes. *jg40897* (*qPFW1.2*) is related to xenla sigma non-opioid intracellular receptor 1, potentially involved in stress responses. Genes such as *jg4542* (*qPFW2.1*) and *jg41157* (*qPH1.1*) are involved in the synthesis of cytokinin hydroxylase, while *jg35082* (*qPH4.2*) and *jg35546* (*qFW4.2*) are associated with cytokinin riboside 5’-monophosphate phosphoribohydrolase, both play roles in cytokinin metabolism. *jg4047* (*qPH6.1*), *jg38379* (*qFW1.1*), and *jg493* (*qDW6.1*) are connected to ethylene-responsive transcription factor WRI1OS, a regulator of ethylene-mediated responses. Additionally, *jg29235* (*qPH4.1*) is tied to abscisic acid 8’-hydroxylase 4, an enzyme crucial for abscisic acid catabolism, while *jg52548* (*qPH3.1*) is associated with abscisic acid receptor, a component of abscisic acid signaling. *jg20169* (*qPH5.1*) is the production of auxin-responsive proteins, and *jg37885* (*qPFW1.1*) is related to brassinosteroid insensitive 1-associated receptor kinase 1, which participates in brassinosteroid signaling.

qRT-PCR was performed to validate expression levels of the candidate genes in bulks with extreme phenotypic traits. Significant differences in expression were observed for 15 candidate genes. The *jg*493, *jg*37885, *jg*5441, *jg*29235, *jg*52548, *jg*35546, *jg*36211, *jg*28336, *jg*20144, *jg*54309 and *jg*53159 exhibited 2.95-fold and 4.47-fold higher expression, respectively, in accessions with larger corresponding trait. The *jg*20169, *jg3*8379, *jg*35082 and *jg*41157 exhibited 2.77-fold and 5.26-fold higher expression, respectively, in accessions with smaller corresponding trait. However, no significant differences in expression were observed for *jg*4047, *jg*40897, *jg*4542 and *jg*38331 between accessions with extreme traits (**Supplementary Figure S1**). These findings provide valuable insights into the genetic mechanisms underlying vetch biomass related traits and highlight potential targets for further functional studies and breeding applications.

### KASP marker development and validation

**Figure 5:**
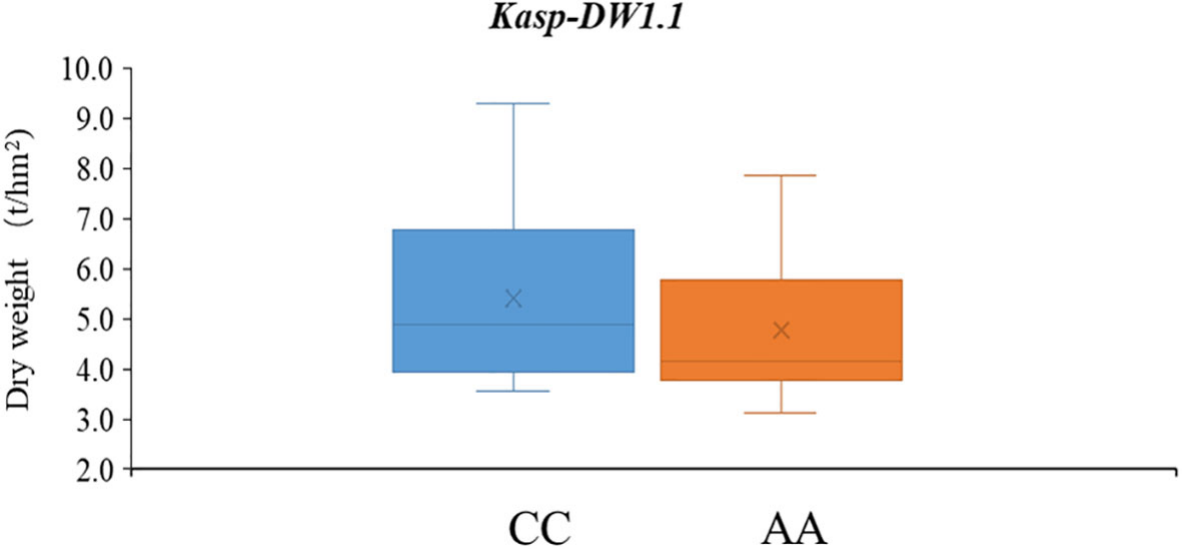
The developed KASP marker *Kasp-DW1.1* in the 172 common vetch accessions.

To support MAS breeding in common vetch, four SNPs with stable or high PVEs were selected to convert into KASP markers. Among these, only SNP within the genetic interval of *qDW1.1* was successfully converted into a functional KASP marker, and named as *Kasp-DW1.1*. Genotypic consistency analysis showed that *Kasp-DW1.1* achieved over 90.1% concordance with resequencing data, confirming its accuracy in reflecting true genotype. Validation in a diverse panel revealed that accessions carrying the favorable allele (CC, 63 lines) had significantly higher DW (mean = 5.40) compared to those with the unfavorable allele (AA, 75 lines; mean DW = 4.74) (*P* < 0.05) (**Supplementary Tables S8**, **S9**; **Figure 5**).

## Discussion

To date, limited research has been conducted on the genetic analysis of biomass related traits, with no molecular markers currently available for breeding practical (De la Rosa and González, 2010). However, significant progress was made in common vetch genetic research following release of the reference genome (Xi et al., 2022), coupled with advancements in resequencing technologies, genotyping arrays, and the development of KASP (Rasheed et al., 2025). This study employed a GWAS approach, utilizing 172 vetch cultivars to investigate the genetic architecture of five biomass related traits.

The 172 common vetch accessions could be divided into 4 subgroups. The subpopulation structure was validated through phylogenetic analysis and population structure. These findings are consistent with previous studies (Xi et al., 2022), which demonstrated that Chinese vetch varieties are broadly divided into two or three distinct groups. Specifically, northwestern populations show closer genetic affinity to European accessions, while southwestern populations exhibit unique adaptive traits (Jia et al., 2021; Xi et al., 2022). The results highlight significant genetic structure associated with geographic origin, offering valuable insights for the development of improved vetch varieties in different regions.

Statistical analysis revealed significant differences in biomass-related traits among the subpopulations, indicating adaptive evolution in common vetch. The POP1 subpopulation exhibited the tallest plants (PH: 113.4 cm) and the highest FW (36.4 t/ha), suggesting that this subpopulation has been naturally selected for enhanced photosynthetic capacity and is likely better adapted to high-fertility and well-watered environments, with greater yield potential. In contrast, the other subpopulations had shorter plants (PH: 78.7-98.3 cm) and lower FW (20.1-22.3 t/ha), which may be due to prioritizing stress resistance (e.g., to drought or wind) or root growth, making them more suitable for environments with biotic and abiotic stresses. In summary, the POP1 subpopulation is better adapted to high-fertility and well-watered environments and has higher yield potential, while the other three subpopulations are more suited to environments with severe abiotic stresses and exhibit better stress tolerance.

We conducted a GWAS on 172 common vetch accessions across four environments to investigate five biomass-related traits. Biomass-related traits in common vetch are typically governed by quantitative inheritance and are controlled by multiple minor genes (Liu et al., 2013; Min et al., 2020). Association mapping was performed using the best linear unbiased predictor (BLUP) values for each trait across all four environments. As a result, all 33 loci identified in this study represent novel findings particularly for the five biomass related traits. For a long time, research bothering on the genetic mechanisms underlying biomass-related traits in common vetch has been limited (Dong et al., 2017; 2019), and there have been no clear reports on relevant genetic loci (Dong et al., 2017; 2019; Rui et al., 2018). Furthermore, this study utilized resequencing and derived SNP markers, which differ from traditional markers such as SSR marker (Chung et al., 2013). As a result, all 33 loci identified in this study represent novel findings. These results provide valuable insights for future research into the genetic mechanisms of biomass-related traits in common vetch.

A total of 15 candidate genes associated with biomass-related traits were identified. Among these, *jg20144* (*qPH5.1*) and *jg53159* (*qPH3.1*) encode the ABC transporters, which are known to be involved in the transport of plant hormones and secondary metabolites and are crucial for regulating plant growth. *jg28336* (*qPFW4.1*) encodes the cellulose synthase, a key enzyme responsible for cellulose synthesis, are essential for the formation of primary and secondary cell walls (Zhao et al., 2021), and influence plant morphology and the overall growth of plants. The *jg54309* (*qPH3.2*), encodes an F-box protein, which are known to regulate hormone signaling, photomorphogenesis, and flowering time (Zhang et al., 2019). Their substrate specificity ensures precise temporal control over developmental transitions and stress adaptation (Dharmasiri et al., 2005; Zhang et al., 2019). *jg5441* (associated with *qPFW2.2*) encodes a serine/threonine-protein phosphatase, which plays crucial roles in cell cycle progression, signal transduction, and hormone signaling by modulating the activity of other proteins (Máthé et al., 2019; Sojka et al., 2024).

Plant hormones centrally regulate diverse aspects of growth and development. Auxins, for instance, are crucial for processes including seed germination, growth and development, flowering, fruit ripening, and leaf abscission (Gomes and Scortecci, 2021; Li et al., 2021). Gibberellins promote stem elongation, break seed dormancy, stimulate germination, and enhance fruit development (Ritonga et al., 2023). Cytokinins primarily promote cell division, delay leaf senescence, and coordinate with auxins to regulate root and bud differentiation. Abscisic acid inhibits growth, promotes dormancy and organ abscission, and accumulates under stress conditions (e.g., drought), inducing stomatal closure to reduce water loss (Brookbank et al., 2021). Ethylene promotes fruit ripening, organ abscission, and senescence, with high auxin concentrations potentially stimulating its synthesis. Brassinosteroids, a more recently characterized class of plant hormones, promote cell elongation and division, enhance stress resistance (e.g., disease and cold tolerance), and may function synergistically with auxins and gibberellins (Gomes and Scortecci, 2021; Zhao et al., 2021; Brookbank et al., 2021; Ritonga et al., 2023). In this context, 10 candidate genes were identified: *jg38379* (*qFW1.1*) and *jg493* (*qDW6.1*) encode ethylene-responsive transcription factors; *jg41157* (*qPH1.1*) and *jg36211* (*qPFW4.2*) encode cytokinin hydroxylases; *jg35082* (*qPH4.2*) and *jg35546* (*qFW4.2*) encode cytokinin riboside 5’-monophosphate phosphoribohydrolases; *jg29235* (*qPH4.1*) and *jg52548* (*qPH3.1*) encode abscisic acid receptors; *jg20169* (*qPH5.1*) encodes an auxin-responsive protein; and *jg37885* (*qPFW1.1*) encodes a brassinosteroid insensitive1-associated receptor kinase.

The main reasons for the failure of KASP primer design in common vetch are as follows: (1) A relatively large proportion of the common vetch genome consists of repetitive sequences. If the SNP sites are located in repetitive sequences, low-complexity regions, or near paralogous genes, it is likely to cause non - specific binding. In addition, high GC/AT content also increases the difficulty of design. (2) A reliable amplification program suitable for common vetch has not been established yet. Currently, the program still mainly refers to that of wheat. It is possible that the settings such as annealing temperature are not reasonable, resulting in unsuccessful amplification. (3) Improper design of primer length or Tm value may lead to ineffective binding or amplification of primers. It may also cause the formation of dimers or hairpin structures. (4) The genome of common vetch was published in 2022, and there is no updated version yet. There may be deficiencies in the genome annotation of common vetch, which leads to the failure of primer design.

The issue of incomplete genotype matching between KASP assays and resequencing results following reasons. Firstly, KASP marker detection is based on allele-specific PCR technology. It uses specific primers and fluorescent probes to distinguish genotypes at specific SNP loci, making it suitable for rapid genotyping of known sites. Resequencing requires fragmenting DNA, constructing sequencing libraries, and using high-throughput sequencing platforms (e.g., Illumina, PacBio, Oxford Nanopore) to sequence the libraries, generating large amounts of raw sequencing data. After removing low-quality reads and adapter sequences, the sequencing reads are aligned to a reference genome (using BWA, Bowtie) to determine their genomic positions. Based on the alignment results, genetic differences between the sample and the reference genome are identified, including SNPs, InDels, SVs (using GATK, Samtools). Secondly, certain SNP loci may be located in repetitive sequences, structural variant regions, or highly polymorphic regions, where KASP primers may fail to bind specifically or amplify effectively. Resequencing can identify variants in these complex regions, providing more accurate genotype information. Thirdly, KASP result interpretation relies on setting fluorescence signal thresholds; improper threshold settings can lead to genotyping errors. Resequencing result interpretation relies on alignment algorithms and variant calling tools; misjudgments can occur due to parameter settings or algorithm biases. Through data comparison, we found that in this study, most inconsistencies were indeed caused by NA (no call) or heterozygotes. Fouth, genomic studies on common vetch are relatively scarce, and its genome contains abundant repetitive sequences and complex regions. These factors may cause bias in KASP primer design or the genotyping process, and affecting result accuracy. Lastly, suitability of the technology system: while KASP technology has been widely and successfully applied in crops like wheat, its application in common vetch is still exploratory.

Application of molecular markers in crop improvement has proven successful. High-throughput and cost-effectiveness are key points for crop MAS and genomic selection (GS). Recently, KASP offers effective and low-cost approach (Liu et al., 2023; Chen et al., 2024; Rasheed et al., 2025). Conversion of SNPs into KASP markers could pave the way for common vetch MAS breeding. Only one SNP successful converted to KASP marker (*Kasp-DW1.1*) and validated in the natural population. Additionally, accessions with more favorable alleles, superior biomass related traits and appropriate agronomic traits, such as GLF295, GLF299, GLF313, GLF321, GLF325, GLF328, GLF329, GLF330, GLF331, GLF335, HZMC1382 and HZMC1487 are recommended as parental lines for improvement of biomass related traits.

## Conclusion

GWAS was performed on 172 common vetch accessions to investigate biomass-related traits. A total of 33 significant loci were identified, with explaining 11.6-22.5% of the PVEs. All identified loci are novel, offering new insights into the genetic basis of these traits. Based on gene annotation, 15 candidate genes were identified and further validated using the qRT-PCR and represent potential targets for functional studies and breeding applications. A KASP marker, *Kasp-DW1.1*, was developed and validated for its utility in vetch breeding programs. We provide stable loci, available KASP markers, and accessions with outstanding biomass related traits that can be directly utilized for molecular breeding and genetic improvement of common vetch.

## Data Availability

The original contributions presented in the study are included in the article/**Supplementary Material**. Further inquiries can be directed to the corresponding authors.

## Supplementary material

Supplementary Figure 1 qRT-PCR for the 19 candidate genes for 5 biomass related traits.
