# Supplementary figures and images for: Genome wide association analysis for biomass related traits in common vetch (Vicia sativa L.)
Source: Front Plant Sci. 2025 Sep 29;16:1647985. doi: 10.3389/fpls.2025.1647985 (PMC12515951; doi:10.3389/fpls.2025.1647985)

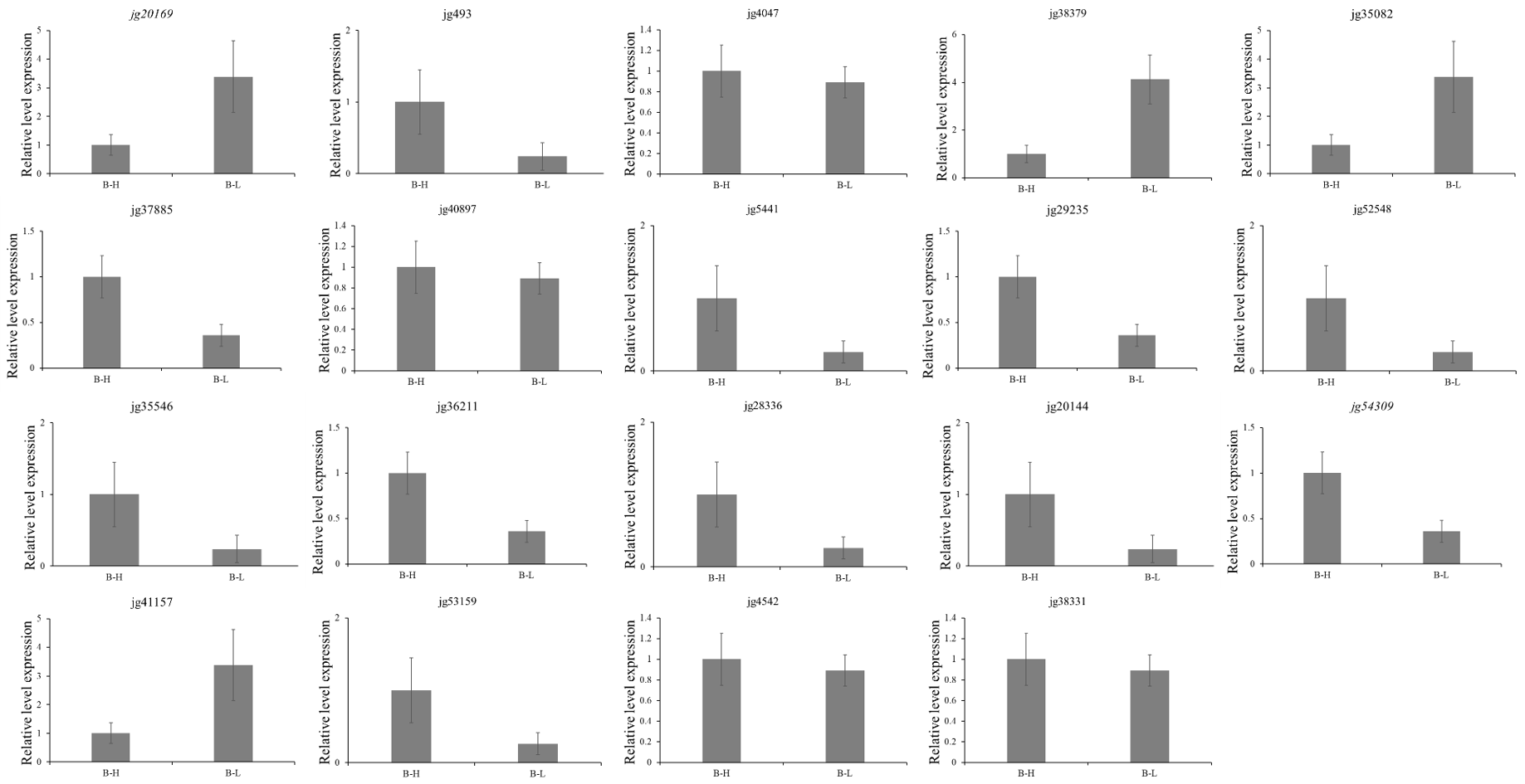

Supplement: Supplementary Figure 1 — qRT-PCR for the 19 candidate genes for 5 biomass related traits. [file Image1.tif]
