# Supplementary material for: Genome wide association analysis for biomass related traits in common vetch (Vicia sativa L.)
Source: Front Plant Sci. 2025 Sep 29;16:1647985. doi: 10.3389/fpls.2025.1647985 (PMC12515951; doi:10.3389/fpls.2025.1647985)
Supplement: Supplementary file 4 [file Table3.docx]

Table S3 The biomass related traits in common vetch among four subgroups.

| Sub-group | Plant height (cm) |  | Plant fresh weight (g) | Plant dry weight (g) | Fresh weight (t/hm^2^) | Dry weight（t/hm^2^） |
| --- | --- | --- | --- | --- | --- | --- |
| POP1 | 113.4^a^ |  | 198.8^a^ | 36.7^a^ | 36.4^a^ | 6.3^a^ |
| POP2 | 87.0^ab^ |  | 180.4^a^ | 37.3^a^ | 20.7^b^ | 3.9^b^ |
| POP3 | 98.3^ab^ |  | 174.6^ab^ | 37.1^a^ | 22.3^b^ | 4.2^b^ |
| POP4 | 78.7^b^ |  | 168.2^b^ | 37.9^a^ | 20.1^b^ | 3.9^b^ |
