# Supplementary material for: Genome wide association analysis for biomass related traits in common vetch (Vicia sativa L.)
Source: Front Plant Sci. 2025 Sep 29;16:1647985. doi: 10.3389/fpls.2025.1647985 (PMC12515951; doi:10.3389/fpls.2025.1647985)
Supplement: Supplementary file 5 [file Table4.docx]

**Table S4** ANOVA for 5 biomass related traits in a panel of 172 common vetch accessions

| **Source of variation** | ***df*** | **MS** | | | | | |
| --- | --- | --- | --- | --- | --- | --- | --- |
|  |  | **PH** | **FW** | **DW** | **PFW** | **PDW** | |
| Genotypes | 171 | *** | *** | *** | *** | *** | |
| Environments | 3 | *** | *** | *** | *** | *** | |
| Replicates | 2 | * | ** | *** | ** | ** | |
| Genotype × Environment | 513 | *** | *** | *** | *** | *** | |
| Error | 1376 |  |  |  |  |  |  |

PH, Plant height; FW, Fresh weight; DW, Dry weight; PFW, Fresh weight per plant; PDW, Dry weight per plant.
