# Supplementary material for: Genome wide association analysis for biomass related traits in common vetch (Vicia sativa L.)
Source: Front Plant Sci. 2025 Sep 29;16:1647985. doi: 10.3389/fpls.2025.1647985 (PMC12515951; doi:10.3389/fpls.2025.1647985)
Supplement: Supplementary file 6 [file Table5.docx]

Table S5 The accessions used for qRT-PCR of the candidate gene for biomass related traits in common vetch

| Number | PH | | PFW | | FW | | DW | |
| --- | --- | --- | --- | --- | --- | --- | --- | --- |
|  | Lower bulk | Higher bulk | Lower bulk | Higher bulk | Lower bulk | Higher bulk | Lower bulk | Higher bulk |
| 1 | GLF313 | GLF307 | GLF529 | HZMC1486 | GLF342-2 | HZMC1485 | GLF295 | HZMC1376 |
| 2 | GLF363 | HZMC1356 | GLF365 | GLF317 | GLF354 | HZMC1362 | GLF340 | HZMC1494 |
| 3 | GLF329 | HZMC1484 | GLF350-2-2 | HZMC1535-1 | GLF335 | HZMC1376 | GLF362 | HZMC1367 |
| 4 | GLF349-1 | GLF373 | GLF349-1 | HZMC1535 | GLF358 | HZMC1383-2 | GLF295-1 | HZMC1381 |
| 5 | GLF321 | HZMC1482 | GLF358 | HZMC1484 | GLF295-1 | HZMC1372 | GLF350-2-1 | HZMC1362 |
| 6 | GLF331 | HZMC1352 | GLF350-2-1 | GLF530 | HZMC1487 | HZMC1367 | GLF354 | HZMC1370 |
| 7 | GLF352 | HZMC1388 | HZMC1382 | GLF330 | GLF370 | GLF309 | GLF335 | HZMC1385 |
| 8 | GLF328 | HZMC1371 | GLF299 | HZMC1358 | GLF295 | HZMC1370 | GLF330 | HZMC1387 |
| 9 | GLF370 | GLF309-1 | GLF367 | GLF334 | GLF325 | HZMC1387 | GLF350 | GLF309 |
| 10 | GLF529 | HZMC1381 | GLF346 | HZMC1488 | GLF340 | HZMC1489 | GLF358 | HZMC1489 |

PH, Plant height; FW, Fresh weight; DW, Dry weight; PFW, Fresh weight per plant; PDW, Dry weight per plant.
