# Supplementary material for: Genome wide association analysis for biomass related traits in common vetch (Vicia sativa L.)
Source: Front Plant Sci. 2025 Sep 29;16:1647985. doi: 10.3389/fpls.2025.1647985 (PMC12515951; doi:10.3389/fpls.2025.1647985)
Supplement: Supplementary file 9 [file Table8.docx]

**Table S8** Polymorphic KASP markers used in this study

| **KASP** | **Physical Position (Mb)** | **Primer Name** | **Sequence (5’to 3’)** |
| --- | --- | --- | --- |
| *Kasp-DW1.1* | 144.3 | FAM | GAAGGTGACCAAGTTCATGC cttgatacacatcatcatctgcatT |
|  |  | HEX | GAAGGTCGGAGTCAACGGAT cttgatacacatcatcatctgcatG |
|  |  | Common | acataacaactcacacgaacaaca |

FAM and HEX tails used for KASP marker assays were indicated in bold.
